# High-Throughput Screening of Biodiversity for Antibiotic Discovery

**Published:** 2018

**Authors:** S. S. Terekhov, I. A. Osterman, I. V. Smirnov

**Affiliations:** Shemyakin–Ovchinnikov Institute of Bioorganic Chemistry, Russian Academy of Sciences, Miklukho–Maklaya Str., 16/10, Moscow, 117997, Russia; Department of Chemistry, Lomonosov Moscow State University, Leninskie Gory, 1, Moscow, 119991 , Russia; Skolkovo Institute of Science and Technology, Skolkovo, Moscow Region, 143026, Russia; National Research University “Higher School of Economics”, Myasnitskaya Str., 40, Moscow, 101000 , Russia

**Keywords:** high-throughput screening, antibiotic discovery, antibiotic resistance, microfluidics

## Abstract

The increasing number of infections caused by antibiotic-resistant strains of pathogens challenges modern technologies of drug discovery. Combinatorial chemistry approaches are based on chemical libraries. They enable the creation of high-affinity low-molecular-weight ligands of the therapeutically significant molecular targets of human cells, thus opening an avenue toward a directed design of highly effective therapeutic agents. Nevertheless, these approaches face insurmountable difficulties in antibiotic discovery. Natural compounds that have evolved for such important characteristics as broad specificity and efficiency are a good alternative to chemical libraries. However, unrestricted use of natural antibiotics and their analogues leads to avalanche-like spread of resistance among bacteria. The search for new natural antibiotics, in its turn, is extremely complicated nowadays by the problem of antibiotic rediscovery. This calls for the application of alternative high-throughput platforms for antibiotic activity screening, cultivation of “unculturable” microorganisms, exploration of novel antibiotic biosynthetic gene clusters, as well as their activation and heterologous expression. Microfluidic technologies for the screening of antibiotic activity at the level of single cells are, therefore, of great interest, since they enable the use of a single platform to combine the technology of ultrahigh-throughput screening, next-generation sequencing, and genome mining, thus opening up unique opportunities for antibiotic discovery.

## INTRODUCTION

**Fig. 1:**
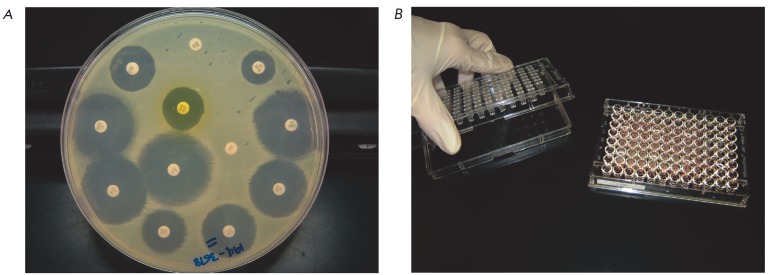
The conventional methods used for antimicrobial activity screening: (*A*) searching for bacterial colonies yielding large zones of inhibition and (*B*) subsequent determination of MICs (adopted from [4])

The discovery of antibiotics was one of the 20th century’s greatest achievements: it increased the survival rate, life expectancy, and quality of life for millions of people. The period between the 1940s and 1960s, when most of the modern antibiotics and their derivatives were discovered, is commonly referred to as “the golden era of antibiotic discovery” [1]. Such impressive results were achieved thanks to the successful combination of an efficient, simple and inexpensive screening platform and the successful selection of the exploration object. This platform, later termed the Waksman platform [2], was based on the cultivation of soil-dwelling bacteria on agar plates. Antibiotic-producing bacteria were identified by covering these plates with an overlay agar layer seeded with the target bacteria, and the candidate clones were detected according to the formation of inhibition zones (*Fig. 1*) [3, 4]. Subsequent screening for the clones producing antibiotics in growth medium was carried out by using the serial dilution procedure and determining the minimum inhibitory concentrations (MICs). Eventually, the discovery of novel antibiotics using the Waksman platform was impeded by the antibiotic rediscovery problem. This platform could detect only culturable and rapidly growing soil bacteria (predominately *Streptomyces*), which could constitutively produce large amounts of antibiotics. Meanwhile, this platform had also made inexpensive and highly efficient natural and semi-synthetic drugs easily available. Hence, the Waksman platform fully aligned with the goals and objectives of its time, since such a problem as the uncontrolled use of antibiotics did not exist during “the golden era of antibiotic discovery.”

The role of antibiotics in nature consists in maintaining the biodiversity of microorganisms resulting from the counteraction of bacteria that produce and degrade antibiotics [5] via various mechanisms [6-8] which are fairly common in various ecological niches [8-11] and had evolved long before human civilizations appeared [12]. The uncontrolled use of large amounts of antibiotics has created unprecedented conditions for the selection and mobilization of resistance genes among bacterial populations and for their subsequent entrapment by the cells of pathogenic microorganisms. The resistance has been evolving via three main mechanisms [13]: primary capture of the resistance genes, mostly through mobilization and horizontal transfer from environmental sources; emergence of compensatory mutations that neutralize the negative effect of the entrapment of resistance genes [14]; and activation of the internal resistance mechanisms, such as active transport [15, 16]. All these factors give rise to the emergence of strains exhibiting multiple drug resistance [17], which is especially typical of the so-called ESKAPE pathogens (*Enterococcus faecium*, *Staphylococcus aureus*, *Klebsiella pneumoniae*, *Acinetobacter baumannii*, *Pseudomonas aeruginosa*, and *Enterobacter *spp.), which are health- and life-threatening [17].

## LIMITATIONS IN USING THE COMBINATORIAL DIVERSITY OF CHEMICAL LIBRARIES IN ANTIBIOTIC DISCOVERY

Combinatorial chemistry and high-throughput screening of chemical libraries have proved efficient for creating drugs targeted at the regulation of various processes taking place in human cells. However, multiple attempts to use high-throughput combinatorial screening to design novel broad-spectrum antibiotics have failed, despite the substantial financial and material investments made and the fact that all the available technologies were used [18-20].

The key reasons for these failures were as follows: Firstly, xenobiotics are not particularly good at penetrating bacterial cells, especially Gram-negative bacteria. Secondly, antibiotics do not obey the Lipinski’s “rule of five” [21]: the physicochemical properties of the combinatorial chemical libraries selected for most drugs are not optimal for antibiotics [22]. Thirdly, the chemical space of the existing libraries is noticeably limited [23]. Meanwhile, the use of chemical libraries enables one to identify various adjuvants, which significantly potentiate the antimicrobial properties of the known antibiotics [24-26], antimetabolites [27], and antivirulence drugs [28] and can also lead to the development of narrow-spectrum drugs specific to a certain target, as demonstrated for bedaquiline, a selective inhibitor of *Mycobacterium tuberculosis *ATP synthase [29]. The creation of specialized chemical libraries targeting an enhanced ability of xenobiotics to penetrate bacterial cells is of utmost importance for the combinatorial methods used to search for novel antibiotics. An alternative strategy is to search for ligands that inhibit the activity of bacterial xenobiotic transport systems.

## SCREENING OF THE NATURAL BIODIVERSITY FOR THE SEARCH FOR NOVEL ANTIBIOTICS

Screening of natural products offers a significantly higher potential for discovering antimicrobial activity [30], probably due to the fact that natural products contain a broader range of stereoselective pharmacophores that have already undergone natural selection for various biological activities over the course of evolution [23]. Metabolomics, which underlies modern approaches for the screening of natural antibiotics [26], uses a combination of tandem separation and analysis techniques, such as high-performance liquid chromatography coupled with mass spectrometry or nuclear magnetic resonance spectroscopy (HLPC-MS or HLPC-NMR, respectively), and whole-genome sequencing methods [31]. Metabolomics makes it possible to proceed to functional genomics [32] and to identify novel ribosomal or nonribosomal peptides [33, 34], as well as secondary metabolites [35].

The range of natural products used to search for antibiotics is rather diverse and includes extracts from plants, fungi, lichens, endophytes, marine plants, seaweeds, corals, and other microorganisms [36]. Nevertheless, it is worth mention that many active substances from these sources have a nonspecific mechanism of membrane destabilization, which, in turn, impedes their application because of the high toxicity caused by a low therapeutic index. Hence, due to the diversity of bacteria and their evolutional propensity to produce antibiotics in order to conquer ecological niches, these organisms still remain one of the most attractive sources of antimicrobial activity. The problem of antibiotic rediscovery can be solved using various approaches.

## STRATEGIES EMPLOYED TO SOLVE THE ANTIBIOTIC REDISCOVERY PROBLEM

The BioMAP platform, which enables the detection of known antibiotics and the discovery of novel ones according to their individual inhibition profiles, has shown that the growth-inhibiting activity of various microorganisms in the collection can be used as a characteristic “fingerprint” of the substance or extract [37]. Collections of bacterial strains of the same species can be used to discover the target of the active substance or, contrariwise, to search for compounds having a specific mechanism of action. A collection of 245 *S. aureus *strains with suppressed gene expression allowed to discover platensimycin, an antibiotic that belongs to a previously unknown class of inhibitors of the enzyme FabF/B that catalyze fatty acid biosynthesis [38].

**Fig. 2:**
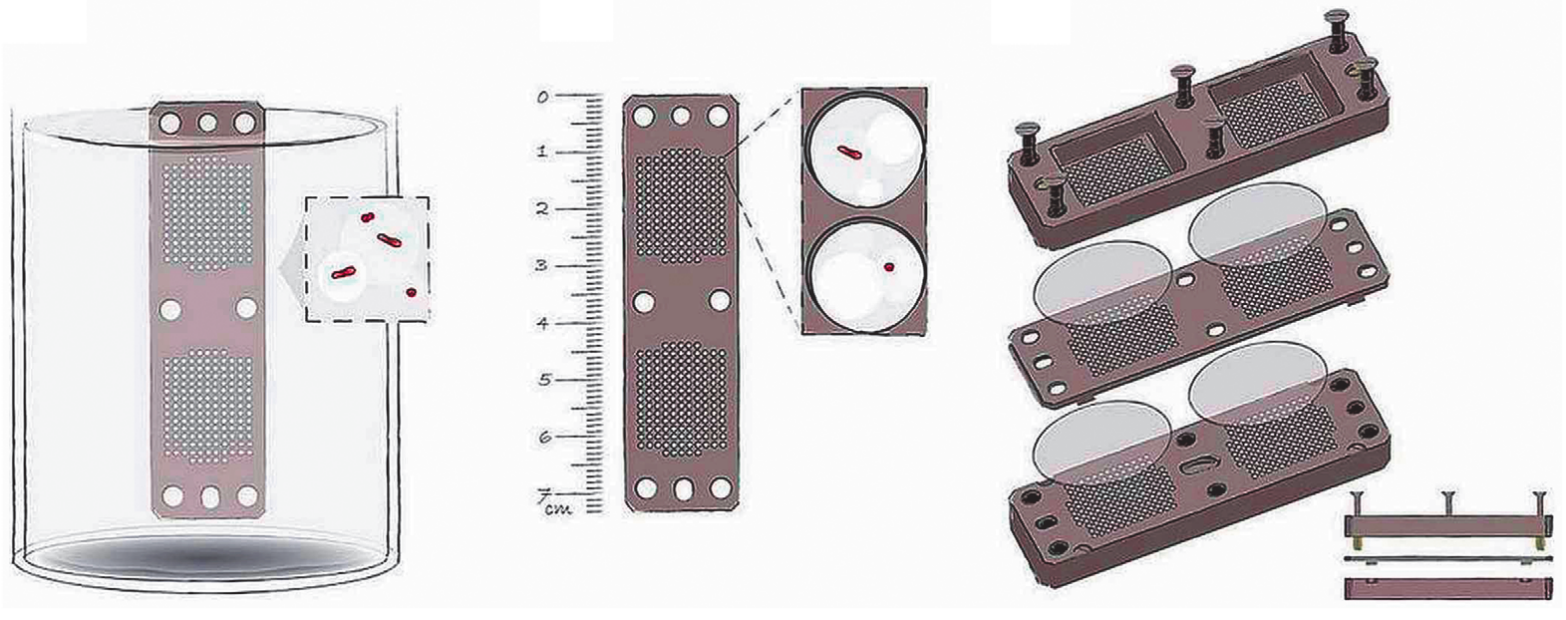
A device for cultivation of “unculturable” soil bacteria (adopted from [47]). The device consists of two compartments separated by a semipermeable membrane. Individual cells of soil bacteria in the growth media are placed on one side of the membrane, while the other compartment contains soil with the required growth factors

The new insight into using soil bacteria as a source of novel antibiotics opens up new possibilities for the screening for antimicrobial activity. Whole-genome sequencing of actinomycetes has shown that they have a much higher ability to produce secondary metabolites upon cultivation. Complete genome sequencing of *Streptomyces coelicolor *has demonstrated that over 20 secondary metabolites can be produced in theory, whereas only three of them have been identified upon *in vitro *cultivation [39]. In its turn, activation of silent genes in antibiotic-producing bacteria opens up new sources of previously unknown antimicrobials [40], while the bioinformatic analysis and gene clustering methods enable *de novo *prediction of antibiotics [41]. Hence, genome mining strategies can be successfully employed to search for novel microbial secondary metabolites, including previously unknown antibiotics that show a high potential for drug design [42]. One of the approaches used to activate silent genes and produce novel antibiotics is to select a growth medium for culturing antibiotic-producing clones which have been pre-selected at the sequencing stage because of the presence of new genes [43]. Using quorum-sensing factors is another approach applied to activate silent genes [44]; however, their effect is difficult to predict, and, therefore, it is probably not always the optimal mechanism for activating silent genes. Meanwhile, recombinant expression is one of the most obvious strategies for activating silent genes [45, 46]. Application of new methods of cultivation of “unculturable” soil bacteria (*Fig. 2*) is another alternative approach that was used to search for novel antibiotics. The platform based on the cultivation of individual soil-dwelling bacteria in their natural environment using a semipermeable membrane has made it possible to discover the novel antibiotic teixobactin, which exhibits activity against resistant strains of Gram-positive bacteria, while resistance to this compound has not developed [47]. Furthermore, this platform allowed to identify the previously unknown genus *Entotheonella*, which is characterized by a unique combination of secondary metabolites and the pathways of their synthesis [48].

Screening of antibiotic-resistant bacteria can be used to reveal novel mechanisms of synergistic interactions, which opens up new prospects for the search for antibiotic adjuvants potentiating their effect [26]. Application of resistant strains has made it possible to discover acyldepsipeptides, a novel class of antibiotics that activate intracellular bacterial protease ClpP [49], which causes the death of bacteria, including persisters, and treats chronic infection [50]. Pre-screening of glycopeptide-resistant soil bacteria increased the probability of discovering clones that produce novel antibiotics belonging to that class by more than 1,000-fold and made it possible to identify pekiskomycin, a novel antibiotic with an unusual scaffold structure [51].

The strategy of designing bifunctional agents acting as ‘Trojan horses’ has also proved efficient. The conjugate of a rifampicin analogue connected to antibodies specific to *S. aureus *cell wall teichoic acids via a biodegradable linker proved efficient in eliminating not only suspension cells, but also the vancomycin-resistant intracellular reservoir of bacteria [52]. It was of crucial importance to select the antibody, linker, and antibiotic properly. Rational design of highly specific antibiotics proved efficient for siderophore–antibiotic conjugates [53].

Proceeding from *in vitro *inhibition to the direct assessment of the antimicrobial activity of an agent *in vivo *opens up new prospects for designing the most efficacious drugs. Screening of antimicrobial activity against *M. tuberculosis *using infected macrophages [54] enabled to make the *in vitro *model as similar to *in vivo *conditions as possible and to rule out compounds that exhibited nonspecific cytotoxicity and low ability to penetrate macrophage cells. Models of *in vivo *infection of the nematode *Caenorhabditis elegans *[55] and the zebrafish *Danio rerio *[56] allowed to select agents that cause the elimination of bacteria, including antibiotics acting via mechanisms that are different from those of the conventional antimicrobial activity.

A high sensitivity of the analytical signal is the fundamental parameter needed to enhance screening performance. Application of bacteria producing recombinant fluorescent reporter proteins as a biosensor of antimicrobial activity makes it possible to directly detect bacterial growth inhibition [57], to identify antibiotics that act via the given translation inhibition mechanism [58], and to screen antibiotic combinations using several fluorescent reporter proteins that have different excitation/emission spectra [59].

## CONCLUSIONS

The search for novel antibiotics has become an urgent task because of the rapid development of antibiotic resistance. The success rate in the screening of chemical libraries is extremely low; this strategy can be efficient mainly when searching for adjuvants and narrow-spectrum antibiotics. Although the Waksman’s platform traditionally used for screening for the antimicrobial activity of microorganisms has been effective in the past, its further application is associated with an extremely high risk of antibiotic rediscovery. It has been estimated that more than 107 different microorganisms need to be screened for every new antibiotic discovery [60]. This problem can be solved by using alternative platforms based on metabolomics, whole-genome sequencing, bioinformatic analysis, recombinant gene expression, and alternative approaches for the cultivation of “unculturable” microorganisms. The fact that physiologically important antibiotics can be discovered within the human microbiome [61] offers new sources for antimicrobial activity screening. The implementation of microfluidic platforms, which allows conversion from a conventional 2D plate-screening platform to emulsion-based 3D screening in isolated microcompartments, is of particular interest. Cultivation of individual cells in emulsion droplets can be used for screening for antibiotic-resistant bacteria [62] or bacteriolytic activity [63]. This alternative approach offers unique prospects for a high-throughput analysis of the activity of broad cell repertoires.

**Fig. 3:**
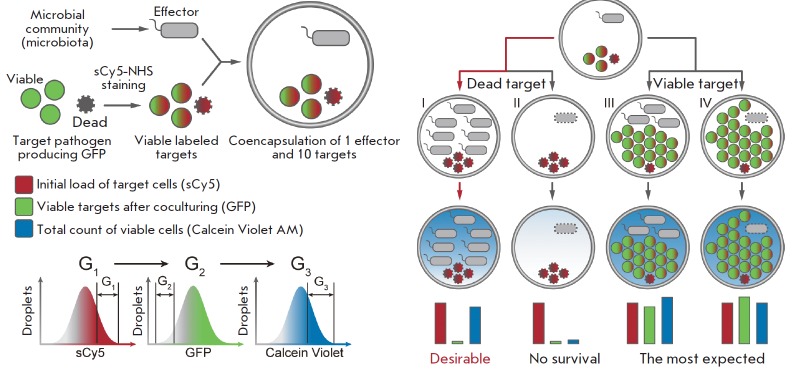
Ultrahigh-throughput (uHT) screening of antimicrobial activity in biocompatible double emulsion droplets (adopted from [64]). Cultivation of single microbiota effector cells with the reporter strain of the target pathogen, followed by intravital staining to detect viable cells with subsequent selection of the target population of effector cells exhibiting antimicrobial activity using FACS

Encapsulation of individual cells into biocompatible double emulsion droplets (*Fig. 3*) enables the analysis of the activity of single cells and the coculturing of representatives of microbiota with target cells to identify antagonistic bacterial strains that produce antibiotics [62]. This method is based on the coencapsulation of individual microbiota species, together with the reporter strain of the target pathogen in droplets of a biocompatible double water-in-oil-in-water emulsion, their subsequent cocultivation in droplets, and FACS-based isolation of the target droplet population where pathogen growth is inhibited, while the effector cells stay viable. The principal advantage of this technology is the fact that the target population of bacterial effectors can be selected, resulting in ultrahigh-throughput (~30,000 cells per second) screening for antimicrobial activity for individual clones. Thus, the selected bacteria can represent a population of extremely rare, slow-growing, and “unculturable” microorganisms, which are, subsequently, identified using whole-genome sequencing, followed by a bioinformatic analysis. This platform was applied for intravital selection of particularly rare cell populations (representing ~0.005%) displaying antimicrobial activity using a single round of screening.

Further development of ultrahigh-throughput (uHT) methods for screening for antibiotic activity is of high interest, since bacterial biodiversity presents a multitude of challenges that require an integrated understanding of the interactions taking place both at the level of individual bacteria and at the level of an entire unique microbiome [65]. The combination of uHT screening and genome mining techniques offers great opportunities for the identification of rare clusters involved in the biosynthesis of microbial secondary metabolites that exhibit different spectra of antimicrobial activity. Such challenges as the analysis of the individual activity of each microbiota species with respect to the given target, as well as extensive assessment of the spectrum of antimicrobial activity against a given microbial community, are of great interest, as they enable one to untangle the interactions that take place within a microbiological community. We believe that advancements in microfluidic technologies, along with uHT screening, whole-genome sequencing, proteomics, and bioinformatics, will further our understanding of microbiological processes. The microfluidic technologies of uHT screening of the natural biodiversity of microorganisms or artificial libraries of antimicrobial compounds clearly has potential for the discovery of the next-generation antibiotics, as well as the selection of ligands that inhibit antibiotic resistance. The combination of these agents may play a crucial role in solving the problem of antibiotic resistance.

## Abbreviations

MIC: minimal inhibitory concentration
ESKAPE: Enterococcus faecium, Staphylococcus aureus, Klebsiella pneumoniae, Acinetobacter baumannii, Pseudomonas aeruginosa and Enterobacter spp.
HPLC-MS: high-performance liquid chromatography coupled with mass spectrometry
HPLC-NMR: high-performance liquid chromatography coupled with nuclear magnetic resonance spectroscopy
BioMAP: antibiotic mode of action profile
FACS: fluorescence-activated cell sorting
GFP: green fluorescent protein
sCy5: sulfo-Cyanine 5
NHS: N-hydroxysuccinimide
uHT: ultrahigh-throughput
